# Coronary artery bypass graft in an isolated single coronary ostium with triple vessel disease

**DOI:** 10.1093/jscr/rjab516

**Published:** 2021-11-30

**Authors:** Nael Al-Sarraf, Baskaran Chandrasekar

**Affiliations:** Department of Cardiac Surgery, Chest Diseases Hospital, Kuwait; Department of Cardiology, Chest Diseases Hospital, Kuwait

## Abstract

Single coronary artery (SCA) is a rare congenital anomaly whereby only one coronary artery arises from single coronary artery ostium of the aortic trunk and supplies the whole heart. The incidence of this anomaly is 0.0024–0.066% in patients undergoing coronary angiography. It is usually an isolated anomaly and majority of patients are picked up incidentally. Lipton classification is widely used to classify this anomaly. Most cases are detected by computerized tomography and/or coronary angiography and are treated medically. Here, we present a rare case of type RII-B in which the patient underwent coronary artery bypass graft for severe triple vessel disease presenting as acute myocardial infarction.

## INTRODUCTION

Single coronary ostium is a rare congenital anomaly that is diagnosed by coronary angiography and/or computerized tomography. Its presentation varies from incidental pick up to sudden death. Treatment is tailored to the presentation and type of anomaly and includes medical therapy, stenting and coronary artery bypass graft surgery.

## CASE REPORT

**Figure 1:**
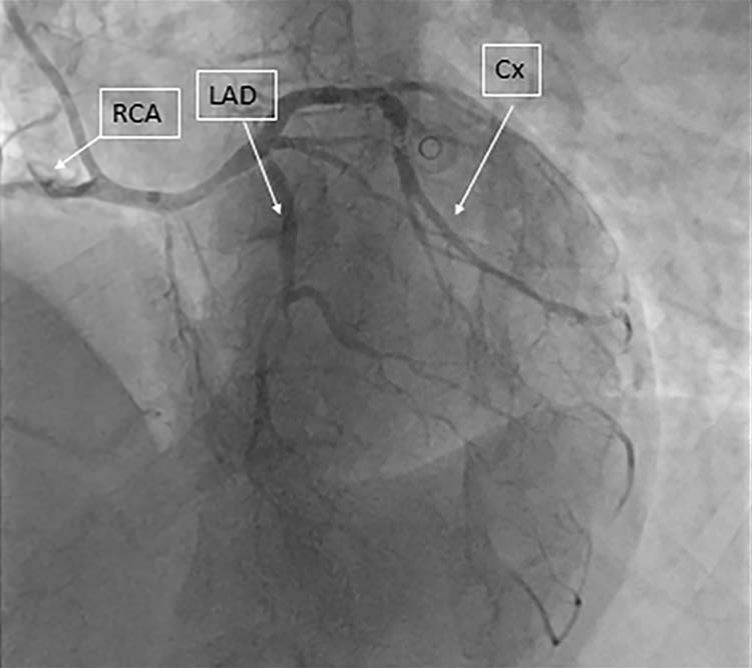
Coronary angiography showing the three coronary arteries arising from single ostium with short LAD and occluded RCA and circumflex artery is shown.

**Figure 2:**
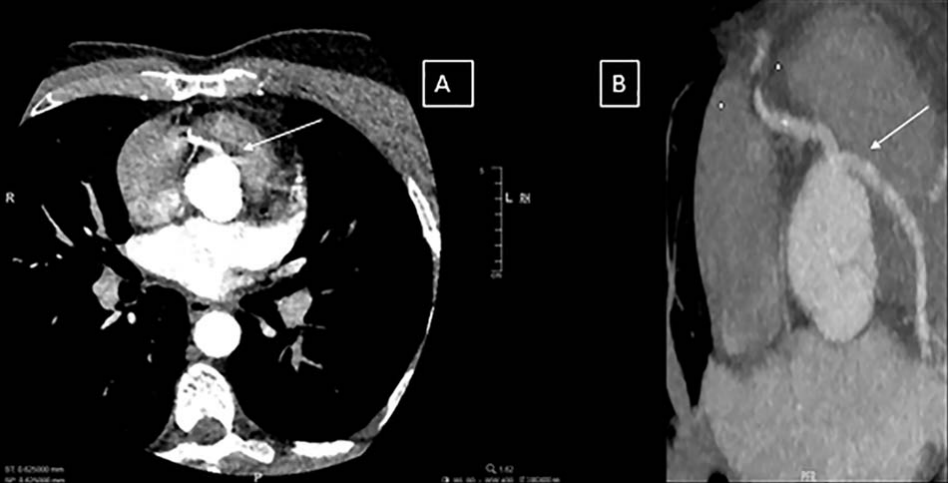
(**A** and **B**) Computerized tomography images showing the single coronary artery ostium and pathway of the abnormal vessel.

A previously fit and healthy 47-year-old man was admitted to his local hospital with delayed infero-lateral ST segment elevation myocardial infarction (STEMI). His surgical history was significant for correction of left forearm deformity since childhood. He had persistent left forearm contracture with significant atrophy in in left arm and left leg. He was treated medically at the time of presentation and transferred to our center for coronary angiography, which showed significant triple vessel disease arising from single right coronary ostium (Fig. 1). Transthoracic echocardiography showed ejection fraction of 40% with hypokinesia of inferior and lateral walls and trivial mitral valve regurgitation with no associated congenital defects. Computed tomography (CT) of the heart (Fig. 2) showed both left main and right coronary arteries arising from one single ostium in the anterior coronary sinus. Left main stem was seen passing between aorta and right ventricular outflow tract in sub pulmonic course. Left anterior descending artery (LAD) was short and had significant stenosis in its ostium. Magnetic resonance imaging (MRI) heart showed no evidence of any cardiovascular congenital anomalies with situs solitus and levocardia with normal arterial trunk connection. As his coronary anatomy was unsuitable for stent insertions, he was referred for coronary artery bypass graft (CABG) surgery. Patient underwent CABG through median sternotomy with left internal mammary artery anastomosed to LAD, reverse saphenous vein graft anastomosed to obtuse marginal and reverse saphenous vein graft anastomosed to posterior descending artery. LAD was deep intra myocardial. Procedure was performed with cardiopulmonary bypass (CPB) and cardioplegic arrest. Patient was weaned from CPB with minimum doses of norepinephrine and was extubated 12 hours post operatively. He was discharged home uneventfully on fifth postoperative day with no complications. On follow-up at 12 months, he remained well with no symptoms.

## DISCUSSION

Single coronary artery (SCA) is a rare congenital anomaly whereby only one coronary artery arises from single coronary artery ostium of the aortic trunk and supplies the whole heart. The incidence is 0.0024–0.066% in patients undergoing coronary angiography [1, 2]. It is more common in men than women with mean age in fifth decade [2, 3]. Majorities of these anomalies are incidental findings. The cause of SCA is uncertain. It may lead to angina, myocardial infarction, arrhythmias, heart failure and sudden death [4]. Myocardial ischemia can be due to acute angle take off of the abnormal vessel with a narrowed slit-like orifice that collapses in a valve-like manner thereby limiting blood flow. Other anatomical features responsible for ischemia are the proximal intramural course of the anomalous vessel, which is squeezed within aortic wall and the compression of the anomalous vessel along its course between aorta and pulmonary artery, particularly during exercise [1]. Approximately, 40% of SCA anomalies are associated with congenital heart defects such as tetralogy of Fallot, transposition of great arteries, coronary arterio-venous fistula and bicuspid aortic valve [1]. However, the majority of cases are isolated [5]. In a series of 4445 patients undergoing CT, only 12 cases with SCA were detected and all were isolated defects. The commonest single ostium was right sinus (11 out of 12) and one only from left. Only two cases had CABG due to severe stenosis and others were treated medically [5].

Coronary angiography is the gold standard investigation. However, CT can provide anatomical information of prognostic value [4]. Lipton classification is widely used [3]. The artery is classified based on the site of origin of the sinus of valsalva into Left (L) or right (R). The anatomical course of the artery is subdivided into types I, II and III. Type I had the anatomical course of either left or right coronary artery (RCA), type II arise from the proximal part of the normal RCA or the left coronary artery and cross the base of the heart before assuming the normal position of the inherent coronary artery. Type III describes the anomaly where the LAD and left circumflex arise separately from the proximal part of the normal RCA. Type II is further subdivided into letters A (anterior), B (between aorta and pulmonary artery) and P (posterior to aorta). Our case was classified as RII-B.

In a review of 10 cases reported by coronary angiography, 70% of SCA were from right sinus of valsalva and there was no atherosclerosis in 70% of patients. All patients were treated medically except for one patient who required stent implantation [1]. Two previous cases were treated medically due to atypical chest pain [4, 6]. However, in our case, the patient had severe multi-vessel disease with acute MI and high-risk anomaly and CABG were the gold standard for his case. Surgical options include osteoplasty, CABG of the anomalous artery and pulmonary artery translocation [7]. Moodie et al. [8] suggested an association of single coronary artery of type RII-B and sudden death during exercise. Originally, this was attributed to compression of left main coronary artery between aorta and pulmonary artery caused by distension of these vessels during exercise. However, another theory postulated was kinking of the left main at its origin from the RCA by increased angulation caused by aortic distension during exercise. Surgical correction and CABG were advocated for this subgroup [2, 8].

## CONCLUSION

In patients with SCA, careful planning of treatment is of paramount importance. The work up of such cases should include CT and/or MRI to define the proper classification of the anomaly and the presence of other associated cardiac anomalies. Treatment is tailored to the extent of disease, clinical presentation and the subtype of coronary anomaly.
